# The dystrotelin, dystrophin and dystrobrevin superfamily: new paralogues and old isoforms

**DOI:** 10.1186/1471-2164-8-19

**Published:** 2007-01-17

**Authors:** Hong Jin, Sipin Tan, Jane Hermanowski, Sabrina Böhm, Sabrina Pacheco, Joanna M McCauley, Marc J Greener, Yaniv Hinits, Simon M Hughes, Paul T Sharpe, Roland G Roberts

**Affiliations:** 1 Division of Genetics & Molecular Medicine, Department of Medical & Molecular Genetics, King's College London, Guy's Hospital, London SE1 9RT, UK; 2 Department of Craniofacial Development, King's College London Dental Institute, Guy's Hospital, London SE1 9RT, UK; 3 MRC Centre for Developmental Neurobiology and Randall Division of Cell and Molecular Biophysics, Guy's Campus, King's College London, London SE1 1UL, UK

## Abstract

**Background:**

Dystrophins and dystrobrevins are distantly related proteins with important but poorly understood roles in the function of metazoan muscular and neuronal tissues. Defects in them and their associated proteins cause a range of neuromuscular disorders. Members of this superfamily have been discovered in a relatively serendipitous way; we set out to compile a comprehensive description of dystrophin- and dystrobrevin-related sequences from available metazoan genome sequences, validated in representative organisms by RT-PCR, or acquired *de novo *from key species.

**Results:**

Features of the superfamily revealed by our survey include: a) Dystrotelin, an entirely novel branch of the superfamily, present in most vertebrates examined. Dystrotelin is expressed in the central nervous system, and is a possible orthologue of *Drosophila *DAH. We describe the preliminary characterisation of its function, evolution and expression. b) A novel vertebrate member of the dystrobrevin family, γ-dystrobrevin, an ancient branch now extant only in fish, but probably present in our own ancestors. Like dystrophin, zebrafish γ-dystrobrevin mRNA is localised to myosepta. c) The extent of conservation of alternative splicing and alternative promoter use in the dystrophin and dystrobrevin genes; alternative splicing of dystrophin exons 73 and 78 and α-dystrobrevin exon 13 are conserved across vertebrates, as are the use of the Dp116, Dp71 and G-utrophin promoters; the Dp260 and Dp140 promoters are tetrapod innovations. d) The evolution of the unique N-terminus of DRP2 and its relationship to Dp116 and G-utrophin. e) A C-terminally truncated common ancestor of dystrophin and utrophin in cyclostomes. f) A severely restricted repertoire of dystrophin complex components in ascidians.

**Conclusion:**

We have refined our understanding of the evolutionary history and isoform diversity of the five previously reported vertebrate superfamily members and describe two novel members, dystrotelin and γ-dystrobrevin. Dystrotelins, dystrophins and dystrobrevins are roughly equally related to each other. Vertebrates therefore have a repertoire of seven superfamily members (three dystrophins, three dystrobevins, and one dystrotelin), with one lost in tetrapods. Most invertebrates studied have one member from each branch. Although the basic shared function which is implied by the common architecture of these distantly related proteins remains unclear, it clearly permeates metazoan biology.

## Background

Dystrophin, identified nearly 20 years ago as the protein deficient in Duchenne muscular dystrophy (DMD)[1], is now recognised as the founder member of a protein superfamily with representatives throughout the animal kingdom[2]. The ~70-kDa signature structure of this family is a compact cluster of domains comprising four EF-hand motifs and a ZZ-domain, followed by a looser region with a propensity to form coiled-coils. In the case of dystrophin and its close paralogue utrophin[3], this structure has a large N-terminal extension of actin-binding CH domains and up to 24 spectrin repeats. The use of internal promoters and alternative splicing of genes encoding both of these proteins enables the generation of multiple N-terminally truncated isoforms of varying length. DRP2 is a further paralogue which retains only two of the spectrin repeats[4]. Dystrophin, utrophin and DRP2 together constitute the vertebrate members of the dystrophin branch of the superfamily; although their function is not well understood, they each (via their signature C-terminal domains) form the core of a membrane-bound complex comprising dystroglycan, sarcoglycans and syntrophins, known as the dystrophin-glycoprotein complex (DGC) [5]. Loss of dystrophin and other components of the DGC causes disorders of the nervous and muscular tissues[6,7]. Loss of utrophin in mouse gives a very subtle phenotype but exacerbates the consequences of dystrophin loss[8]. The consequences of DRP2 deficiency are unknown.

Dystrobrevins, also part of the DGC, show ~30% identity to dystrophins, and form the other branch of the superfamily. These lack the long N-terminal extension of the dystrophins, and are little longer than the superfamily signature structure itself. Vertebrates have two paralogous dystrobrevins, α- and β-dystrobrevin [9-12]. α-dystrobrevin has a characteristic C-terminal extension and a range of isoforms which rivals dystrophin's; loss of function results in a phenotype similar to that found in dystrophin mutants[13]. Combined loss of α- and β-dystrobrevin results in a combination of skeletal myopathy and behavioural disorders[14].

Invertebrates, as is the case with many gene families, have a simpler repertoire of proteins; all metazoans so far examined (including, for example, the fruit fly and the nematode) have a single dystrophin and a single dystrobrevin[15,16]. Mutant phenotypes so far described tend to be of a synaptic form; loss of dystrophin or dystrobrevin in nematodes results in a hypersensitivity to acetylcholine[17], while dystrophin deficiency in flies alters the dynamics of neurotransmitter release at the neuromuscular junction[18].

Dystrophins and dystrobrevins interact directly with each other via homologous C-terminal coiled coil motifs[19,20] and depend strictly on each other for their localisation and incorporation into the DGC[14]. It seems apparent that this heterodimeric partnership has arisen through an ancient duplication (prior to the divergence of metazoan phyla) of a single gene encoding a simple homodimeric protein[2], followed by structural and functional differentiation of the two proteins. The increased complexity of the vertebrate body has brought with it the need for further diversity of the repertoire of DGCs, such that there are three paralogous dystrophins with a combined total of at least 15 isoforms, two dystrobrevins with at least eight isoforms, five syntrophins instead of two, and six sarcoglycans instead of three.

We set out to assess the diversity of dystrophin- and dystrobrevin-related sequences throughout metazoa using a combination of bioinformatic analysis and experimental methods. Our data enable us to clarify the serial duplication of the single invertebrate dystrophin gene to give rise to the vertebrate dystrophin, utrophin and DRP2 genes; we also reveal the existence in fish (and almost certainly in our own ancestors) of a third dystrobrevin gene. We catalogue the extent of alternative splicing and alternative promoter use across vertebrate dystrophins and dystrobrevins, and we find that ascidians have an unexpectedly simplified DGC. Perhaps most significantly, we report the discovery of an entirely new branch of the protein superfamily, dystrotelin, which is as distantly related to the dystrophins and dystrobrevins as they are to each other.

## Results and discussion

Starting from known dystrophin- and dystrobrevin-related sequences, our principal approach was to use publicly available genome databases as a starting point from which to establish the likely extent of isoform- and paralogue-based diversity. Predictions based on this were then tested experimentally and supplemented by acquiring further data that were not predictable from the databases. Confirmed sequences were subjected to further bioinformatic analysis and, in the case of dystrotelin, to preliminary functional and expression analysis.

### DRP2

DRP2 is a small vertebrate paralogue of dystrophin and utrophin, whose expression is largely confined to punctate structures in the CNS[21] and patches between the Cajal bands of schwann cells in the PNS[22]. Its role in the CNS is unknown, but in the PNS DRP2 interacts with L-periaxin via its spectrin repeats, and is reduced in a periaxin-deficient myelination disorder[22].

We compiled a set of sequences which is likely to encompass much of the DRP2 diversity present in extant organisms. Much of this was done by BLAST searches for obvious paralogous exons in available genomic and expressed sequence databases. In addition to this, certain critical sequences had to be obtained experimentally by 5'cRACE[23], namely the 5' end of a bony fish DRP2 transcript (*Danio rerio *[GenBank:DQ443728]), and the missing 40% of a cartilaginous fish DRP2 (*Scyliorhinus canicula *[GenBank:U43517], updated). In order to establish whether the cartilaginous fish was the most divergent organism to possess DRP2, we also made extensive unsuccessful attempts to isolate DRP2-related sequences from cyclostomes (see section on cyclostomes).

**Figure 1:**
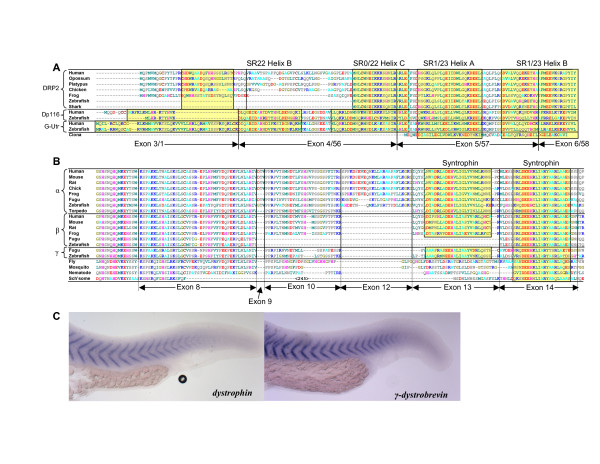
**DRP2 and vertebrate dystrobrevins**. A) Relationship between DRP2, Dp116 and G-utrophin. Alignment of N-terminal DRP2 sequences from a range of animals, together with selected dystrophin Dp116 and G-utrophin sequences. Accession numbers: *H. sapiens *DRP2, [GenBank:U43519]; *Ornithorhyncus anatinus *DRP2, [GenBank:BK005803]; *G. gallus *DRP2, [GenBank:DR425253]; *X. tropicalis *DRP2, [GenBank:CX826467];*D. rerio *DRP2, [GenBank:DQ443728]; *S. canicula *DRP2, [GenBank:U43517]; *H. sapiens *Dp116, [GenBank:NM_004014]; *D. rerio *Dp116, [GenBank:DQ788695]; *H. sapiens *G-utrophin, [GenBank:BP282211]; *D. rerio *G-utrophin, [GenBank:DQ788696]. Other sequences were predicted from publicly available genome data. Yellow boxes indicate helical regions (PSIPRED) annotated with spectrin repeat (SR) numbers for dystrophin and DRP2[24] where applicable. Exon numbers for dystrophin and DRP2 are given [4, 81]. B) Alignment of alternatively spliced region of selected dystrobrevin sequences. All full-length coding exons (numbering after mouse α-dystrobrevin, Ambrose *et al*., 1997 [33]) have been included (the status of the 9-bp exon 9 is not known for frog and torpedo α-dystrobrevin; exon 11 is only used in a C-terminally truncated α-dystrobrevin isoform). Exon boundaries only apply to vertebrate genes; the region corresponding to exons 8–14 is encoded by a single exon in the four invertebrates. Yellow boxes indicate syntrophin binding sites [39]. Accession numbers as follows; α-dystrobrevins: *H. sapiens*, [GenBank:NM_001390]; *M. musculus*, [GenBank:AF143543]; *R. norvegicus*, [GenBank:XM_226109]; *X. laevis *[GenBank:BC046265];*D. rerio*, [GenBank:DQ516343] and [GenBank:DQ516344]; *T. californica*, [GenBank:L06945]; β-dystrobrevins:*H. sapiens*, [GenBank:Y15722]; *M. musculus*, [GenBank:Y15742]; *R. norvegicus*, [GenBank:BC081889]; *D. rerio*, [GenBank:DQ516345]; *D. rerio *γ-dystrobrevin, [GenBank:DQ516346]; *D. melanogaster *dystrobrevin, [GenBank:AF277387]; *C. elegans *dystrobrevin, [GenBank:NP_490860]; *S. mansoni *dystrobrevin, [GenBank:DQ431251]. Other sequences were predicted from publicly available genome and EST data. C) Results of *in situ *hybridisation of probes complementary to the 3'UTRs of zebrafish dystrophin and γ-dystrobrevin transcripts. Wholemount 24 hpf embryos are shown, anterior to the left. The dystrophin staining of vertical myosepta, comparable to that previously described [32], is similar to that obtained with γ-dystrobrevin.

Comparison of most of the DRP2 sequence yields the unremarkable findings expected of a highly conserved vertebrate protein (72% identity, 83% similarity between human and fish). The only point of interest is the DRP2-specific elaboration of the N-terminus and its relationship to its paralogues Dp116 and G-utrophin (Figure 1A). An identifiable orthologue of the first human coding exon (exon 3, encoding residues 1 to 39) was evident in eutherian mammals (dog, cow, mouse, rat), a marsupial (opossum), a monotreme (platypus), a bird (chicken) and an amphibian (xenopus). Although the similarity at the genomic level was marginal for the last two species, these were both supported by ESTs ([GenBank:DR425253] and [GenBank:CX826467], respectively). Outside the tetrapod clade, it is striking that DRP2 sequences only share high homology from human methionine 79 (see Figure 1A). The exonic region immediately 5' of this in three species of fish (*D. rerio*, *Fugu rubripes *and *Tetraodon nigroviridis*) also shows a highly conserved (but fish-specific) sequence. In order to clarify whether Met79 is the first in-frame ATG in non-tetrapods, we performed 5'cRACE on DRP2 RNA from both the bony fish *D. rerio *(zebrafish; whole animal [GenBank:DQ443728]) and from the cartilaginous fish *S. canicula *(lesser spotted catshark; brain [GenBank:U43517], updated). In each case, Met79 was the 5'-most in-frame methionine, preceded by at least one in-frame stop codon (within exon 4 for *S. canicula *and in exon 3 for *D. rerio*). This suggests that Met79 is the ancestral DRP2 start site, and that the tetrapod-specific 49–78-amino acid N-terminal region is a later acquisition, presumably with a specific function. However, initiator codon usage in any DRP2 sequence is still to be confirmed experimentally, and usage may be tissue-specific.

Clear similarity between the DRP2s and other members of the dystrophin family begins at Met79, a residue which is conserved in all vertebrate dystrophins, utrophins and DRP2s and all known insect dystrophins (the N-terminally truncated sea squirt dystrophin starts 22 residues further on; see "Ascidians" below). DRP2s possess two full spectrin repeats (as defined by Winder *et al*[24] and by the SMART database) paralogous to repeats 23 and 24 of human dystrophin, preceded by sequence equivalent to most of the last helix of repeat 22. The gross structural resemblance between DRP2, dystrophin isoform Dp116, and utrophin isoform G-utrophin has been noted before[4,25], and it is clear from the similar sizes and reading frame phase of DRP2 exon 4 and dystrophin and utrophin exon 56 (see Figure 1A) that this entire exon was duplicated during the ancestral formation of the DRP2 gene, and that the sequence upstream of the ancestral DRP2 start site (Met79) has subsequently diverged. It is also possible that the first exon of Dp116/G-utrophin (which lies in intron 55 of the dystrophin and utrophin genes) was also duplicated in this event, and gave rise to the first coding exon (exon 3) of the DRP2 gene, though no similarity is expected to remain. It is notable that there is still some residual similarity at the amino acid level between the first exons of Dp116 and G-utrophin (Figure 1A and [see Additional File 1]).

### The cyclostome dystrophin/utrophin ancestor

In order to resolve the point of divergence of the three paralogous vertebrate dystrophins (dystrophin itself, utrophin and DRP2), we acquired samples from three cyclostomes (the Atlantic hagfish, *Myxine glutinosa*, the sea lamprey, *Petromyzon marinus*, and the brook lamprey, *Lampetra planeri*). We used the pan-dystrophin degenerate primers described previously[4] to perform RT-PCR on total brain RNA. This gave a single dystrophin/utrophin-like sequence in each species, despite various attempts to isolate paralogous sequences (the same RT-PCR performed on mouse brain RNA yields a mixture of paralogues). These failed attempts included a) use of degenerate RT-PCR primers capable of amplifying most vertebrate and many invertebrate dystrophin-related sequences, b) trying to bias the results of this RT-PCR towards DRP2 by digesting the cDNA with rare-cutting restriction enzymes targeted at the respective dystrophin/utrophin sequences, c) use of degenerate RT-PCR primers designed to recognise sequences specifically present in all vertebrate DRP2s, and d) the use of samples from three different cyclostome species (*P. marinus*, *L. planeri *and *M. glutinosa*). While such negative results are necessarily weak, our widespread success at amplifying much more divergent sequences[15] encourages us to conclude that the cyclostomes do not express DRP2.

The *M. glutinosa *and *P. marinus *sequences were further extended by 3'RACE, yielding the natural stop codon ([GenBank:DQ440977] and [GenBank:DQ440978], respectively; the *L. planeri *sequence, [GenBank:DQ440979], was very similar to that of *P. marinus*, as expected, and was not extended). The cyclostome sequences were aligned with the most divergent gnathostome sequences (dystrophin, utrophin and DRP2 from human and shark), with amphioxus dystrophin[15] as an outgroup, and phylogenetic trees were generated (data not shown). The cyclostome sequences consistently diverge after the duplication leading to DRP2, and tend to branch before the divergence of dystrophins and utrophins. This is consistent with the failure to detect a separate utrophin in cyclostomes, and suggests that the gnathostome dystrophin-like molecule is a descendent of the last common ancestor of dystrophin and utrophin. All trees strongly support cyclostome monophyly, as has been repeatedly found with molecular data[26]. We conclude that the possession of distinct dystrophin and utrophin molecules is a specifically gnathostome trait, arising from a duplication that occurred after our divergence from cyclostomes. The duplication which gave rise to the DRP2 gene occurred before this point in time, but after the divergence from amphioxus; thus the last common ancestor of cyclostomes and gnathostomes had two dystrophin-like molecules, namely DRP2 and a common ancestor of dystrophin and utrophin. We assume that the cyclostomes have subsequently lost their DRP2 gene (see above).

The cyclostome sequences share an unsurprising set of mosaic ancestral and derived traits. The only features that warrant mention are the presence of exon 71 sequence, and the absence of a substantial chunk of the conserved C-terminus. The short exon 71 of mammalian dystrophin is alternatively spliced[27,28] and has homologues in all gnathostome dystrophins and utrophins (where it is also alternatively spliced[29]) but not in DRP2s or invertebrate dystrophins. Its presence in the cyclostome sequences shows that it arose after the duplication that gave rise to DRP2 but before the last common ancestor of cyclostomes and gnathostomes. The cyclostome dystrophin/utrophin sequences cease to be alignable with other members of the dystrophin family (or even with each other) soon after the beginning of exon 76 (from human dystrophin residue 3610). This means that they lack not only the 39-residue "ΔE78" C-terminus[30] that is present in all gnathostome dystrophins and most invertebrate dystrophins, but also a further ~70 residues which are conserved in all dystrophins, utrophins and DRP2s. The function of this missing region (human dystrophin residues 3610–3685) is unknown, but clearly dispensable in cyclostomes.

### Dystrobrevins

Most invertebrate metazoan genomes encode a single dystrobrevin-related molecule; the only exception that we found to this was the two ascidians (sea squirts) *Ciona intestinalis *and *Ciona savignyi*, neither of whose genome sequences contains a dystrobrevin-like gene (see "Ascidians" below). Most vertebrates, on the other hand, appear to have two dystrobrevins, as has been previously described[9,10], named α- and β-dystrobrevin. This distinction is clear down to cartilaginous fish, where there are single examples of each type in sequence databases; *Torpedo californica *sequence [GenBank:L06945] [12] is an α-dystrobrevin, while *Squamus acanthias *EST [GenBank:CV670957] is a β-dystrobrevin. In addition to these two vertebrate dystrobrevins, we note, to our knowledge for the first time, the existence in bony fish of a third dystrobrevin, which we name γ-dystrobrevin.

While examining the public databases for fish dystrobrevin-related sequences, we found that all three completed teleost genomes (*D. rerio*, *F. rubripes *and *T. nigroviridis*) showed evidence for three distinct dystrobrevin genes, rather than two. One of these was clearly α-dystrobrevin, while the remaining two seemed superficially to resemble β-dystrobrevin. In phylogenetic analyses, one of these tended to cluster with other β-dystrobrevins (despite appearing to lack exons 10–13 in the syntrophin-binding region), while the other failed to cluster with any known vertebrate dystrobrevins, suggesting that it was a novel paralogue. We verified the transcripts of all three dystrobrevins by RT-PCR and sequence analysis from *D. rerio *whole animal RNA ([GenBank:DQ516343], [GenBank:DQ516345] and [GenBank:DQ516346]); this also clarified and confirmed the exon structure. We named the novel paralogue γ-dystrobrevin by extrapolation from established nomenclature; it is well supported in several fish species by ESTs and is often mis-annotated as β-dystrobrevin (e.g. [GenBank:AY423026]).

A more refined phylogenetic analysis (which deliberately excluded the variable exons 9–13) clearly shows γ-dystrobrevin to represent an independent branch of the dystrobrevin family, separating from β-dystrobrevin earlier than the divergence of fish and tetrapods. The formation of three separate clusters (α-, β- and γ-dystrobrevin) is supported in 100% of bootstrap trials, and branching of γ-dystrobrevin *prior *to the divergence of β-dystrobrevin and α-dystrobrevin is also robustly supported (99% of trials). This suggests that the last common ancestor of fish and tetrapods also had all three dystrobrevins, and that γ-dystrobrevin has been lost secondarily by tetrapods (rather than it arising in a teleost-specific duplication event, as is the case with many other genes[31]). Like the β-dystrobrevins, the γ-dystrobrevins share the presumed ancestral state in lacking the α-dystrobrevin-specific C-terminal tyrosine-rich tail and exons 9 and 12. Apart from general sequence divergence, they have no particular features which differentiate them from the β-dystrobrevins. It is intriguing that while the fish β-dystrobrevins have lost exons 10–13, the γ-dystrobrevins have retained the structure characteristic of tetrapod β-dystrobrevins (see Figure 1B).

In order to assess the gross expression of γ-dystrobrevin, we performed *in situ *hybridisation of *D. rerio *probes complementary to the 3'UTRs of both dystrophin and γ-dystrobrevin. In 26 hours post fertilisation (hpf) embryos these two probes showed virtually indistinguishable patterns of hybridisation to the vertical myosepta (somite boundaries; see Figure 1C), a pattern that has previously been reported for dystrophin[32]. Probes to neither α- nor β-dystrobrevin yield this pattern (data not shown), suggesting that γ-dystrobrevin, like dystrophin, has a specific role in the myoseptum. It also tallies with the fact that a survey of the representation of the seven *D. rerio *superfamily members in the GenBank EST database shows that dystrophin and γ-dystrobrevin are by far the most abundant, with 34 and 33 hits, respectively (compared with 14 for utrophin, 0 for DRP2, 3 for α-dystrobrevin, 2 for β-dystrobrevin and 8 for dystrotelin), reflecting the preponderance of muscle tissue in this animal.

### Alternative promoter use and splicing

**Figure 2:**
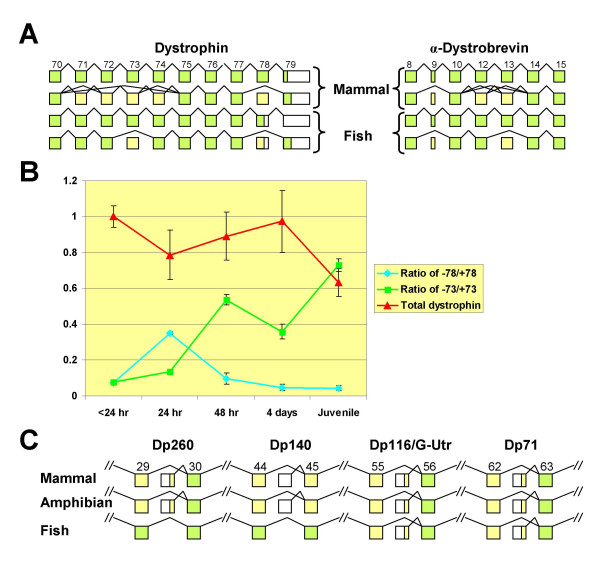
**Alternative splicing and alternative promoter usage**. A) Conservation of alternative splicing of dystrophin and α-dystrobrevin across vertebrates. Mammalian alternative splicing [11, 27–29, 33] and exon numbering [33, 81] are as published. Our data show alternative splicing of dystrophin exons 73 and 78 and α-dystrobrevin exons 9 and 13 in fish. Green boxes – coding regions in each isoform, yellow boxes – skipped coding exons, white boxes, 3'-UTR. B) Developmental profile of dystrophin alternative splicing in zebrafish, showing a steady increase in transcripts lacking exon 73 and a defined peak of transcripts lacking exon 78 early in development. Results are means of three experiments, ± SD. C) Conservation of alternative internal promoter use in the vertebrate dystrophin and utrophin genes. The schematic diagram shows the positions of alternative first exons for the Dp260, Dp140, Dp116 and Dp71 dystrophin isoforms and for G-utrophin, all previously characterised in mammals. Our findings show that Dp260 and Dp140 are conserved across tetrapods and that Dp116, Dp71 and G-utrophin are conserved across all gnathostomes. For each isoform, green boxes show sequence which is protein-coding in both that short isoform and the full-length protein, yellow ones show sequence which is coding in only one of these two, and white shows non-coding sequence. Numbers at top give exon numbers according to the human dystrophin gene [81]. Novel frog and fish sequences are presented separately [see Additional File 2].

The mammalian dystrophin and α-dystrobrevin genes generate an impressive array of protein isoforms through the elaborate use of alternative splicing[11,27-29,33], alternative promoter use [25,34-38] and alternative transcription termination[33]. We set out to determine, by bioinformatics and RT-PCR, whether these isoform-generation mechanisms are conserved across the vertebrate clade (see schematic diagrams in Figure 2A and 2C, and sequence alignments in [Additional File 1] and [Additional File 2]).

#### Alternative splicing of dystrophin exons 71–74

Exons 71–74 undergo complex alternative splicing (omission of exon 71, of exon 74 and of exons 71–74) in humans and mice [27-29]. This has been proposed[39] to regulate the stoichiometry of syntrophins in the dystrophin complex by removing their binding site(s). When we assessed by RT-PCR whether the same occurs in zebrafish, the only alternative splice event seen in this region was the omission of exon 73 (Figure 2A; as previously observed[40]). Amplification of a product spanning exons 70–74 from whole animal RNA from different developmental stages of zebrafish showed this to be temporally regulated, such that that the inclusion of exon 73 drops from > 90% in early embryos to < 60% in juvenile fish (Figure 2B). Thus the ability to regulate syntrophin stoichiometry is regulated across vertebrates, but by slightly different mechanisms. Surprisingly, given that exon 71 is also alternatively spliced in utrophin[29,41], it appeared not to be in zebrafish.

#### Alternative splicing of dystrophin exon 78

Alternatively spliced exon 78 (the penultimate exon of the dystrophin gene) is a vertebrate-specific innovation. In its absence (in mammalian Δ78 transcripts[27,28]), a highly conserved open reading frame, normally in the 3'UTR of exon 79, is brought into frame, conferring an amphipathic alpha-helical C-terminus homologous to the constitutive C-terminus of most invertebrate dystrophins[30]. Exon 78 itself is poorly conserved, and in bony fish even contains an in-frame stop codon (Figure 2A). We tested whether the temporally regulated alternative splicing of exon 78 is conserved across vertebrates by performing semi-quantitative RT-PCR (in a reaction spanning exons 77–79) of whole animal RNA from different developmental stages of zebrafish. This showed a peak of exon 78 omission (25% of dystrophin transcripts) in 24-hour embryos with > 90% inclusion in earlier and later samples (Figure 2B). This temporal pattern shows a striking resemblance to that described by Bies *et al*[27], whereby exon 78 is predominantly excluded in 14-day mouse embryos and predominantly included in adult mice (their Figure 2). In order to establish potential determinants of such alternative splicing, we obtained publicly available sequences of the introns surrounding exon 78, and also used whole genome vectorette PCR to acquire the equivalent sequence from the (more divergent) cartilaginous fish, *S. canicula *[GenBank:DQ641922]. Comparison of these sequences shows little significant conservation save in the environs of the exon 78 donor splice site, where a conserved motif (AGAGgttrgt) surrounds an atypical consensus sequence (position +3 is T in only 2% of vertebrate splice sites [42]; [see Additional File 2, panel A]).

#### Alternative splicing of α-dystrobrevin exons 9–13

Studies in human and mouse have found consistent tissue-specific alternative splicing of exons 12 and 13 of α-dystrobrevin [11,33], a phenomenon which has been shown to afford a facility for regulating the stoichiometry of syntrophins[39], as there are syntrophin-binding sites in exon 13 and exon 14 (these are roughly analogous to exons 72–74 of dystrophin). We found by RT-PCR between exons 8 and 15 of α-dystrobrevin from whole animal zebrafish RNA that exon 12 was present in all products obtained, the tiny 9-bp exon 9 was present in most, but that exon 13 was missing from the vast majority of transcripts (~90%; data not shown, see Figure 2A). This pattern seemed not to vary significantly with developmental stage. This shows that, as with dystrophin, the ability to modulate syntrophin stoichiometry is conserved. As with dystrophin exon 78, we observe that all available α-dystrobrevin (and β-dystrobrevin) exon 13 genomic sequences have an unusual pyrimidine at the +3 position in the donor splice site [see Additional File 2, Panel B]. No alternative splicing of this region was seen in zebrafish β- or γ-dystrobrevin at any developmental stage.

#### The dystrophin Dp260 promoter

Dp260 is an isoform encoded by exons 30–79 of the mammalian dystrophin gene. It is expressed largely, but not exclusively, in the retina[35,43], and its disruption is associated with electroretinopathy in DMD patients[44]. The Dp260 promoter lies just upstream of exon 30 of the human dystrophin gene, and we find that the N-terminal sequence which the corresponding first exon contributes to the Dp260 isoform is highly conserved down to amphibia (see Figure 2C and [Additional File 1, Panel A]), as is the adjacent genomic sequence and the size of the isoform-specific intron (101–130 bp between Dp260 exon 1 and exon 30). The sequence is not conserved in fish, and indeed zebrafish intron 29 is only 113 bp long. Thus the canonical Dp260 isoform[35] seems to be specific to tetrapods and absent from fish. This is at odds with the extremely strong 260-kDa band observed by Bolaños-Jiménez *et al*[40] (their Figure 5), which may therefore arise through a distinct but convergent mechanism. We confirmed the existence of an amphibian Dp260 transcript, predicted from the *Xenopus tropicalis *genome sequence, by RT-PCR from *X. laevis *RNA ([GenBank:DQ831004]; [see Additional File 1, Panel A]).

#### The dystrophin Dp140 promoter

Dp140 is a dystrophin isoform expressed throughout the CNS[36]. The Dp140 promoter lies between exons 44 and 45 of the human dystrophin gene, and although the corresponding first exon splices onto exon 45, the first in-frame initiation codon is not until exon 51 (though there are multiple AUGs in other reading frames). We found that the human, dog, mouse, chick and frog dystrophin genes fulfil the following criteria: a) the first methionine of Dp140 (human dystrophin Met2461), together with its adjacent amino acid sequence in exon 51, is conserved, b) there are no in-frame AUG codons between the start of exon 45 and this methionine, and c) a ~2-kb region of intron 44, including the first exon of the Dp140 transcript, is conserved. By contrast, the zebrafish dystrophin gene a) has no such methionine codon in exon 51 (indeed, in zebrafish, fugu and tetraodon, exon 51 is much shorter than in tetrapods, lacking much of the "hinge 3" region between spectrin repeats 19 and 20), b) there are multiple in-frame methionines in exons 45–50 (three in zebrafish, seven in fugu) and c) not only does intron 44 lack the conserved region, but it is only 234 bp long. Thus, based on sequence homology alone, it seems that the Dp140 isoform is likely to be conserved down to amphibia, but not fish (Figure 2C). We confirmed the existence of an amphibian Dp140 transcript, predicted from the *X. tropicalis *genome sequence, by RT-PCR from *X. laevis *RNA ([GenBank:DQ831006]; [see Additional File 1, Panel B]).

#### The dystrophin Dp116 promoter

Dp116 is a dystrophin isoform expressed largely in the PNS and CNS [34,38]. The Dp116 promoter lies between exons 55 and 56 of the human dystrophin gene, and we find that the N-terminal sequence encoded by the first exon is conserved from mammals down to zebrafish, although different initiation codons may be used in the different species (Figures 1A and 2C, and [see Additional File 1, Panel C). Dp116 transcripts are poorly represented in dbEST, perhaps because PNS as a tissue is underrepresented. We confirmed the existence of a fish Dp116 transcript by RT-PCR from *D. rerio *whole animal RNA [GenBank:DQ788695]. We note the unconfirmed stop codon in the chicken genomic sequence of Dp116 exon 1.

#### The dystrophin Dp71 promoter

Dp71 is expressed at high levels in many tissues [37,38]. The Dp71 promoter lies between exons 62 and 63 of the human dystrophin gene (these being the exons that encode the WW domain), and the N-terminal sequence which the corresponding first exon contributes to the Dp71 isoform is conserved from mammals down to cartilaginous fish (Figure 2C and [see Additional File 1, Panel D]). The Dp71 transcript from many species is well represented in dbEST, and has been described in mammals[37,38] and zebrafish[40]. Other than noting a single EST extending the conservation to cartilaginous fish [GenBank:CV798132], we include it here merely for completeness.

#### The G-utrophin promoter

G-utrophin is a short isoform analogous in structure to Dp116 and DRP2, expressed in the brain and sensory ganglia[25]. Although there are very few transcribed G-utrophin sequences in the databases, the first exon (and the strongly α-helical peptide sequence that it encodes) is exceptionally well conserved across vertebrate genomes. We confirmed the existence of a fish G-utrophin transcript by RT-PCR from *D. rerio *whole animal RNA [GenBank:DQ788696]. There is sufficient residual sequence similarity (at the amino acid level) between this first exon and that of the Dp116 transcript to suggest that they derive from duplication of a single ancestral first-exon sequence (Figures 1A and 2C and [see Additional File 1, Panel C]). We further note the existence in the zebrafish genome (but not in the other completed fish genomes) of a G-utrophin pseudogene which comprises counterparts of almost all exons from G-utrophin exon 1 to exon 71. Although many of these exons have aberrant splice sites and have incurred many missense mutations, it is still actively transcribed; there is an EST [GenBank:BI982568] which contains exons 1, 56 and 57, and we were able to amplify a frameshifted cDNA containing exons 64, 68, 69, 70 and 71 ([GenBank:DQ831005]; [see Additional File 1, Panel C]).

### Streamlining in ascidians

Genome sequence is available for the ascidians (sea squirts) *Ciona intestinalis *and *C. savignyi*; in addition we have previously obtained a partial dystrophin-like sequence from *C. intestinalis *by degenerate RT-PCR[15]. Examination of the urochordate genomes enables a fuller assessment of their complement of dystrophin complex components. The dystrophin sequence is encoded by a single exon in each species, and is highly idiosyncratic ([GenBank:AK173373] for *C. intestinalis*). The length is a mere 802 residues in *C. intestinalis *and 842 in *C. savignyi*, these being ~75% identical to each other. These proteins have only two spectrin repeats (orthologous to dystrophin repeats 23 and 24), followed by the dystrophin-like series of WW, EF and ZZ domains; in this respect they grossly resemble vertebrate Dp116 or DRP2 (see Figure 1A). However, C-terminal to the ZZ domain the sequence becomes extremely degenerate, with few recognisable similarities to the vertebrate dystrophins. This is surprising for members of phylum chordata, given the obvious strong homology in this region between vertebrate dystrophins and dystrophins from other phyla (molluscs, arthropods, echinoderms and nematodes, for example[15]).

Both *Ciona *species have a second dystrophin-related molecule of a similar size and domain to dystrobrevins and dystrotelins (*C. intestinalis *[GenBank:BR000175]. This is similarly conserved (71% identity) between species, and on phylogenetic analysis shows a strong tendency to cluster with vertebrate dystrotelins (see below). Another ascidian, *Molgula tectiformis*, has a related, albeit shorter, sequence ([GenBank:CJ352374] and [GenBank:CJ403437]) which also clusters with the dystrotelins.

In line with the degenerate C-terminus of their dystrophin molecules, there is no evidence for dystrobrevin- or syntrophin-related sequences in either ascidian genome, despite their clear detectability in much more distantly related genomes. There also seemed not to be any sarcoglycan-related sequences, though these are much harder to detect at such evolutionary distances. Of the core dystrophin complex, therefore, only a severely N-terminally truncated and C-terminally degenerate dystrophin, a dystroglycan, and a dystrotelin-like molecule were detectable. It seems likely that as part of its derived biological streamlining[45,46], the ascidian has dispensed with much of its dystrophin complex. Incidentally, the *C. intestinalis *dystrophin and dystrotelin sequences were identified as "zinc-finger genes" by Miwata *et al*. (their Ci-ZF345 and Ci-ZF346), and subjected to expression analysis by *in situ *hybridization on developing embryos[47]. Unfortunately neither gene yielded distinct patterns at any developmental stage.

### The dystrotelin family

**Figure 3:**
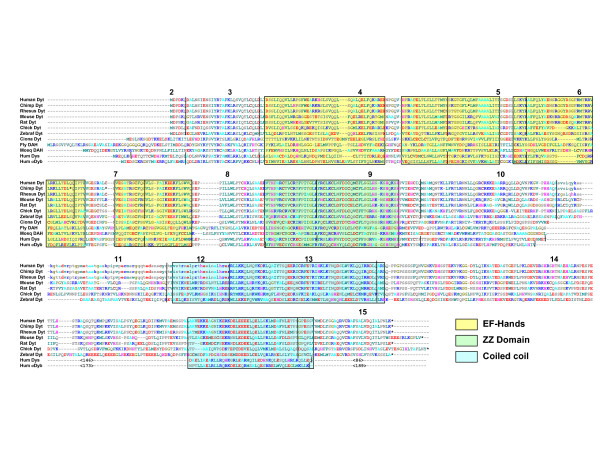
**The dystrotelins and DAHs**. Alignment of full-length sequences of dystrotelins ("Dyt") from a range of vertebrate species. Those from *H. sapiens*, *M. musculus *and *D. rerio *have been confirmed by RT-PCR and sequence analysis; the remainder are predicted from genomic sequence alone. Also included for context are the N-terminal sections of *C. intestinalis *dystrotelin-like protein, two insect DAH sequences, human dystrophin (Dp71 isoform; "Dys") and human α-dystrobrevin ("αDyb"; much of the C-terminal regions of these proteins are not alignable with dystrotelins and were omitted). Vertical lines indicate exon boundaries (the invertebrate genes are intronless). Lower case sequences in the primates indicate known (human) or expected (chimp, rhesus monkey) exclusion of the exons from the mRNA. Colour-coded boxes indicate known structural motifs – EF hands [48], ZZ domain [82] and coiled-coil regions predicted by COILS. Note three nonsense mutations (asterisks) in chimp sequence. Accession numbers: *H. sapiens *dystrotelin, [GenBank:DQ516347]; *M. musculus *dystrotelin, [GenBank:DQ443727]; *D. rerio *dystrotelin, [GenBank:DQ443726]; *C. intestinalis *dystrotelin, [GenBank:BR000175]; *D. melanogaster *DAH, [GenBank:AAC24159]; *H. sapiens *Dp71, [GenBank:M92650], *H. sapiens *α-dystrobrevin, [GenBank:NM_001390].

During this study, we noted the existence in most vertebrate genomes of exons potentially encoding a novel, extremely divergent member of the dystrophin/dystrobrevin family. We name this dystrotelin, after its apparent evolutionary distance from the other members of the family (ancient Greek, τήλε, distant). There were very few ESTs corresponding to any dystrotelin from any species, and none that are appropriately spliced. In order to confirm the expression and correct splicing of this gene, we concentrated our efforts on human, mouse and zebrafish, and were readily able to amplify full-length dystrotelin cDNA from brain mRNA of these three species. We present an alignment of known and predicted dystrotelin sequences (Figure 3).

**Figure 4:**
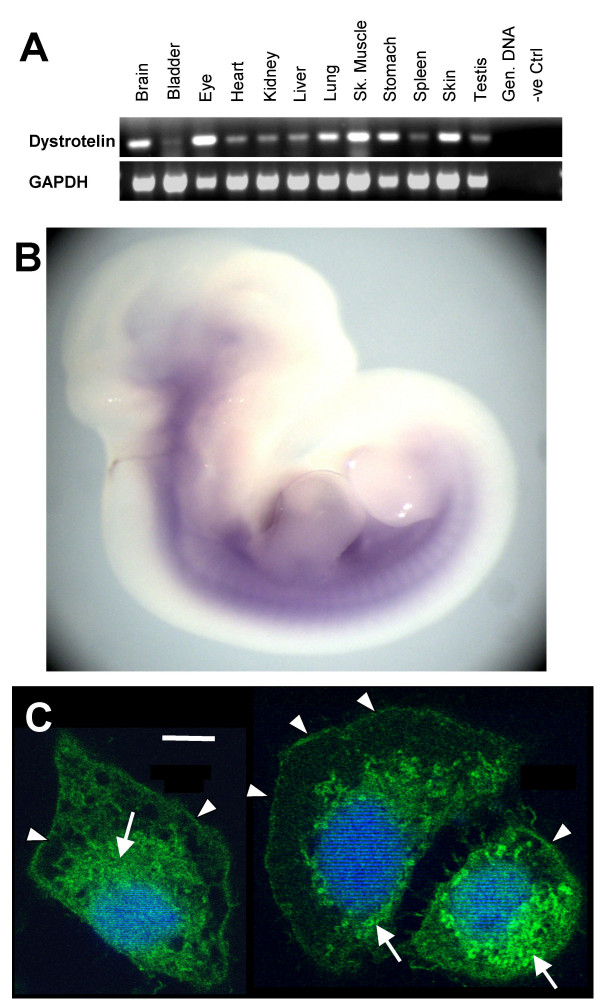
**Preliminary characterisation of mouse dystrotelin**. A) Expression profile of mouse dystrotelin by semi-quantitative RT-PCR of RNA from dissected mouse tissues. GAPDH RT-PCR is given as an RNA loading control. B) *In situ *hybridisation of a dystrotelin cRNA probe to whole-mount mouse embryo (E10.5), showing staining of the central nervous system. C) Confocal fluorescent microscopy of a mouse dystrotelin-EGFP fusion construct heterologously expressed in COS7 cells (arrow-heads, cytoplasmic membrane; arrows, perinuclear staining), compared with the largely nuclear staining observed with the empty EGFP vector. Scale bar 10 μm.

The mouse gene is the best characterised, and will be described in the most detail here. It comprises 15 exons, the first of which is non-coding. The remaining 14 exons encode a protein of 654 amino acids and calculated molecular weight of 74 kDa [GenBank:DQ443727]. The mouse dystrotelin gene maps to chromosome 1, band C2. Although transcripts could be amplified from a broad range of tissues, exon 4 appears to be excluded from the majority of transcripts in tissues other than the brain, giving rise to a frameshifted transcript. Semi-quantitative RT-PCR of in-frame transcripts containing exon 4 (i.e. those with a continuous open reading frame) from adult mouse RNA samples confirmed this stronger expression in nervous and muscular tissues (Figure 4A). *In situ *hybridization of part of the dystrotelin transcript to E10.5 mouse embryos showed that embryonic dystrotelin is largely expressed in the brain and neural tube (Figure 4B)

The human gene has a very similar structure, except that two of the exons, despite being recognisably conserved, have poor or absent splice sites (exon 11 has no upstream polypyrimidine tract, and exon 12 has a GT-to-AT mutation in its donor splice site); accordingly we find these to be absent from amplified human cDNA [GenBank:DQ516347], resulting in an in-frame loss of 75 codons. These apparent splice site defects are also present in chimp and rhesus monkey, where we anticipate the splicing behaviour to be similar. The resulting interstitial deletion may have little functional impact on the protein, however, as exon 11 is very poorly conserved and exon 12 is very small. The primate dystrotelin genes have also suffered insertion of a DSCR3 pseudogene in intron 9. The ~70-kb human dystrotelin gene maps to 2q33.3, just distal to the ADAM23 gene (207,224,590-207,291,365 in Ensembl v39).

The zebrafish gene has an identical structure to its mammalian counterparts. The entire coding region [GenBank:DQ443726] is readily amplifiable from whole-animal cDNA of all developmental stages.

The dystrotelin protein shows significant similarity to dystrophin (30% identity, 49% similarity) and dystrobrevin (30% identity, 46% similarity) over the first 270 amino acids, corresponding roughly to the EF hands and ZZ domain. As there is 32% identity, 53% similarity between dystrophin and dystrobrevin over the same region, the three families of proteins appear to be equally related to each other (see Figure 3). This level of similarity is such that the N-terminal region of dystrotelin is expected to form a compact globular tertiary structure grossly indistinguishable from that of the corresponding region of dystrophin[48]. Residues involved directly in dystrophin's interaction with β-dystroglycan[48] [PDB:1EG4], however, are not conserved, making it unlikely that dystrotelin interacts with this ligand.

The C-terminal domain of the dystrotelins (the ~370 amino acids that follow the ZZ domain, i.e. ~60% of the protein) is extremely divergent, to the extent that virtually no residual similarity is detectable between the mammalian and fish dystrotelin C-terminal domains, even using a dotplot with lax parameters. Despite this, the exon structure (length and phase of exons) is absolutely conserved, strongly supporting identity-by-descent, and COILS predicts that coiled-coil structures are favoured in analogous regions of mammalian and fish dystrotelins (see Figure 3). Because of the rapid rate of divergence of the dystrotelins, it is not possible to say whether the C-terminal domain was derived from the C-terminus of the dystrophin-like ancestor; there is no residual statistically significant similarity. However, a region predicted to form coiled-coils (residues 569–604 of mouse dystrotelin) contains a motif with striking resemblance to the second coiled-coil region of dystrophins and particularly dystrobrevins (compare, for example, human dystrotelin IKERKDELEEE with human α-dystrobrevin LRQRKDELEQR), which suggests identity-by-descent or convergent evolution. Secondary structure prediction by PSIPRED[49] yields surprisingly coherent structures for mammalian and fish dystrotelin C-termini despite the low sequence similarity (like dystrophins and dystrobrevins, these are largely α-helical and unstructured coil).

The C-terminal coiled-coil-forming regions of dystrophins and dystrobrevins have been shown to associate with each other[19,20], an association which we have replicated using the yeast two-hybrid (Y2H) technique (data not shown). To test whether the C-terminal region (residues 258–654) of mouse dystrotelin could associate with either of these proteins, we cloned it into the Y2H DNA-binding domain vector pBHA and co-transformed it into L40 yeast with an activation domain construct (based on vector pGAD10) encoding the C-terminal domains of mouse dystrophin, mouse α-dystrobrevin or mouse dystrotelin. None of these three combinations resulted in activation of reporter genes, unlike the co-transformation of dystrophin and α-dystrobrevin, which gave strong activation of reporter genes, reflecting the well-known interaction[19,20]. We conclude that, at least under the conditions of our Y2H assay, dystrotelin is unable to heterodimerise with members of the dystrophin or dystrobrevin families, or to homodimerise.

In order to assess the likely subcellular localisation of dystrotelin, we cloned the entire mouse dystrotelin coding sequence N-terminal to the EGFP coding region of pEGFP-N2 (Clontech) and transfected it transiently into COS-7 cells. EGFP fluorescence was then examined using confocal fluorescent microscopy; this showed most of the dystrotelin-EGFP fusion protein to be localised to the cytoplasmic membrane and to perinuclear structures (Figure 4C). The latter showed substantial overlap with endoplasmic reticulum (ER) staining, but not with mitochondrial staining (data not shown). Collection of a z-stack of confocal images (data not shown) showed this pattern to apply throughout the vertical depth of the cells. Localisation to the ER is a common observation during heterologous overexpression in eukaryotic cells, and may be artefactual. EGFP expressed from the vector alone showed almost entirely nuclear localisation (data not shown). We conclude that heterologously expressed dystrotelin localises spontaneously to the cytoplasmic membrane, and possibly to the ER.

It is clear that dystrotelin is evolving rapidly compared with other members of the dystrophin family. Ka/Ks, the ratio of non-synonymous to synonymous mutations, can act as a measure of the direction and strength of selective pressure acting on a DNA sequence via its ability to encode a protein. We compared the Ka/Ks for vertebrate members of the dystrophin/dystrobrevin/dystrotelin family (except for the γ-dystrobrevins). This gave Ka/Ks < 0.2 within the vertebrate clade and Ka/Ks < 0.1 between mammals for dystrophin, utrophin, DRP2, α-dystrobrevin and β-dystrobrevin, indicative of strong purifying (negative) selection [see Additional File 3]. In contrast, the overall Ka/Ks for the full-length dystrotelin coding region was 0.6–0.7, an unusually high figure for a protein-coding gene. Knowing that the second half of the protein (C-terminal to the ZZ domain) is more variable than the first half, we divided the open reading frame accordingly and reassessed the Ka/Ks. While the first half gives a lower Ka/Ks of 0.4, the second half gave a Ka/Ks of approximately unity; this is usually taken to indicate a lack of selective pressure or a mosaic of positive and negative selection. Because there are manifestly a number of residues within the C-terminal domain which show strong conservation, we favour the explanation that other residues are conversely under positive selective pressure to change. Such pressure is often associated with gene products involved in the immune, reproductive and cognitive systems[50,51].

It is puzzling that whereas human and rhesus monkey (*Macaca mulatta*) have complete dystrotelin open reading frames, the Ensembl sequence for chimp (*Pan troglodytes*) shows the presence of nonsense mutations in three consecutive exons. The surrounding exonic and intronic sequences are otherwise virtually identical to human. We set out to check whether this was perhaps due to poor local sequence quality in the Ensembl sequence by performing genomic PCR on our own chimp and gibbon (*Hylobates lar*) DNA samples; this confirmed all three nonsense mutations in the chimp [GenBank:DQ640072], but showed that the gibbon [GenBank:DQ640073], like human and rhesus monkey, has an intact open reading frame. It therefore appears that, bizarrely, three separate nonsense mutations have become fixed in the chimp dystrotelin gene during the last 7 million years. This shows that dystrotelin function is dispensible for at least one vertebrate.

The origin of the dystrotelin sequence is unclear. We attempted to establish this by using the N-terminal region (which is clearly shared between all dystrophins, dystrobrevins and dystrotelins) to perform a phylogenetic analysis against our most diverse sequences. All tree methods used gave the same, well-supported result, namely that all dystrophins (including those from the nematode and the schistosome) cluster together, as do all dystrobrevins, to the exclusion of the dystrotelin clade. As exon boundaries are slow to change during evolution, we compared the positions of exon boundaries between the alignable region of dystrophins, dystrobrevins and dystrotelins (see Figure 3A). By this reckoning, dystrophin shares one character uniquely with dystrotelin (the position of the E7/E8 boundary) and one with dystrobrevin (the fusion of E5 and E6), while dystrotelin and dystrobrevin share the absence of a WW domain. This approach therefore fails to resolve the trichotomy.

**Figure 5:**
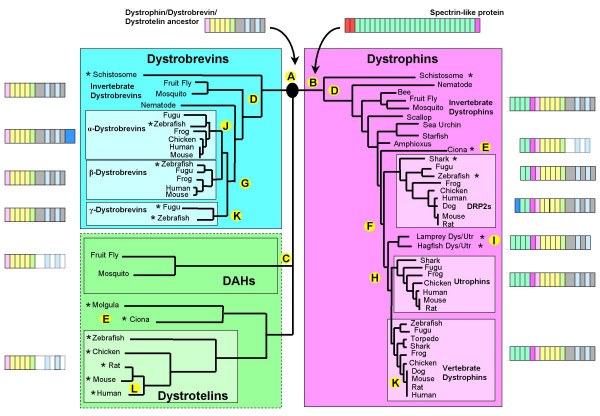
**Phylogenetic tree of the dystrophin/dystrobrevin/dystrotelin superfamily**. This summary tree reflects our best estimate of the evolutionary relationships between members of this protein family. Large coloured boxes – major families within the superfamily (the broken line reflects the tentativeness of the orthology of DAHs and dystrotelins). Lighter boxes – vertebrate family members. Asterisks – sequences described for the first time in this study. Black circle – unresolved point of divergence between dystrophins, dystrobrevins and dystrotelins. Phylogenetic distance is represented by the horizontal axis only. This summary tree has been compiled using a selection of smaller trees based on longer sequences (which are each only applicable to the local branches) and a large tree based on the short sequences which are alignable between all family members. A selection of methods has been used, and the tree shown reflects the consensus topology, using distances from neighbour-joining trees. The only points of equivocation were the position of the cyclostome dystrophin/utrophin branch, the position of the γ-dystrobrevin branch, and the relationship between the DAHs and dystrotelins; the former two are described at length in the text; the latter is left as a trichotomy. Letters A-L are a key to events described in our Conclusions. Schematic diagrams at left and right show domain structures of respective proteins, not drawn to scale.

The implication of this is that the divergence of dystrotelin pre-dates the divergence of schistosomes from other metazoa, and possibly even pre-dates the ancestral duplication that gave rise to the separate dystrophin and dystrobrevin families. If this is so, then dystrotelins should be detectable in invertebrates. We could find no other dystrophin/dystrobrevin paralogues in the nematode or schistosome genomes, but three ascidians have a paralogue which shows some affinity to the vertebrate dystrotelins (see above). In addition, three insects (*Drosophila melanogaster*, *D. pseudoobscura *and *Anopheles gambiae*) have genes encoding DAH (discontinuous actin hexagon[52]), which shows a similar range of attributes to the vertebrate dystrotelins and the urochordate paralogues. Dystrotelins and DAH share the following properties: a) a reasonably well-conserved N-terminal series of domains shared with the dystrophins and dystrobrevins (four EF hands and a ZZ domain), b) a high rate of sequence divergence (fly and mosquito DAH share ~30% sequence identity, compared with ~70% between dystrophins of these two species; these relative rates of divergence are comparable to those for dystrotelin and dystrophin between mouse and zebrafish), c) a very poorly constrained C-terminus with the propensity to form two coiled-coil structures, d) a fairly tight size-range of 640–670 amino acids, e) deep phylogenetic branching outside all known metazoan dystrophins and dystrobrevins (see Figure 5). In summary, given the rapid sequence divergence in these proteins, it is conceivable that dystrotelins and DAH are orthologues, but rather hard to prove.

## Discussion

We present here a comprehensive description of dystrophin- and dystrobrevin-related sequences from available metazoan genome sequences, validated in representative organisms by RT-PCR, or acquired *de novo *by us. The work establishes the breadth of sequence diversity and identifies two novel vertebrate family members (see Figure 5). The implications are many.

The alternative splicing that has previously been reported in mammalian dystrophin and α-dystrobrevin serves to determine the presence or absence of the syntrophin binding regions and of the ancestral C-terminus. However it has been unclear whether this is an arcane mammalian feature or a more widespread phenomenon; indeed many alternative splicing events barely seem to be conserved between mammals [53-55]. Here we show that the temporally regulated alternative splicing of dystrophin exons 73 and 78 is conserved in zebrafish, as is alternative splicing of α-dystrobrevin exons 13 and (to a lesser extent) 9. These suggest that the need to modulate both syntrophin stoichiometry and the presence of the ancestral dystrophin C-terminus are vertebrate-wide, necessitating mechanisms that have been conserved for over 500 million years. A potential basis for the conserved splicing behaviour is noted in a sub-optimal splice donor site at all vertebrate dystrophin exon 78 and α-dystrobrevin exon 13 sequences [see Additional File 2]; in each case these splice sites have a pyrimidine at base +3, normally seen in < 4% of vertebrate donor sites[42]. Furthermore, pyrimidine +3 was also seen adjacent to all β-dystrobrevin exon 13 sequences, which are also alternatively spliced[9], but not γ-dystrobrevin exon 13, which is not alternatively spliced (this work; [see Additional File 2]). This suggests that either specific mechanisms recognise this unusual splice site in conditions when that exon is to be included or, as has been hypothesised, that alternatively spliced exons tend to have sub-optimal splice sites so that they become more dependent on the binding of exonic splice enhancers by conditionally expressed SR proteins.

Similarly, the ability of the vertebrate dystrophin and utrophin genes to generate N-terminally truncated isoforms by the use of internal promoters has only been studied in mammals (except for the description of fish Dp71[40]). In the case of Dp260, Dp140, and G-utrophin, relatively few studies have been performed, and it was until now entirely conceivable that mammal-specific cryptic promoters are responsible for generating transcripts of incidental significance. We find that on the contrary, Dp116 and G-utrophin promoters are as broadly conserved as the Dp71 promoter, with striking levels of conservation throughout vertebrates at the amino acid and nucleotide level. The similarity between Dp116, G-utrophin and DRP2 suggests that there has been a promoter in this intron since before the serial gene duplications which gave rise to the paralogues; indeed the somewhat smaller sea urchin dystrophin isoform SuDp98 arises within the orthologous intron (though using an internal translation initiation site)[56], as does the rather larger *Drosophila melanogaster *dystrophin isoform Dp205[57], suggesting that the promoter is ancient. The situation regarding the dystrophin Dp260 and Dp140 promoters is slightly different; although we here show by their existence in frog that they are probably conserved throughout tetrapods, they are clearly absent in the three available fish genomes, where the corresponding introns, large in tetrapods, are tiny. This tallies with the observation of Neuman *et al*. that intragenic promoters in the dystrophin gene correlate with large introns[57]. It is possible that the Dp260 and Dp140 promoters are more ancient but have been lost in teleosts, which are in many respects highly derived organisms; examining cartilaginous fish might resolve this. It is interesting that wherever the alternative first exon encodes protein, the sequence of the latter is highly conserved [see Additional File 1], implying specific functional importance rather than merely a means of making a truncated protein.

Our studies of DRP2 show that the unique N-terminus is specific to tetrapod DRP2 molecules. This N-terminus is encoded by the first coding exon (exon 3) and the first three-quarters of exon 4. In both bony and cartilaginous fish, translation seems to initiate at a methionine (equivalent to human Met79) near the end of exon 4. In the case of bony fish, there is a conserved ORF upstream of this point, but the preceding exon has an in-frame stop codon; it is therefore formally possible that a different isoform, using a different upstream exon, might give rise to a longer protein than the one we present here. In cartilaginous fish, on the other hand, there is a stop codon a mere five codons upstream of this methionine, so no longer protein can be produced.

Despite the absence of the N-terminal sequence in non-tetrapod DRP2s, we note a weak similarity between the N-termini of DRP2s, Dp116 and G-utrophin, including the presence of several conserved cysteine residues and other short motifs. Whereas the N-termini of Dp116 and G-utrophin are almost certainly descended from a single ancestral sequence (see above), it is conceivable that the similarities with the tetrapod DRP2 N-terminus have arisen by convergent, rather than divergent, evolution. The apparent conserved cysteines (often in the context Q/NXC) suggest to us the possibility of post-translational modification at the N-terminus of all of these proteins, though there are no recognised consensus motifs for such modification.

Cyclostomes, which occupy a taxonomic position intermediate between protochordates and gnathostomes, also show an intermediate elaboration of the dystrophin family, with a single dystrophin/utrophin-like molecule. The point of divergence of this sequence (see Figure 5) strongly suggests that the last common ancestor of cyclostomes and gnathostomes would also have had a DRP2 protein, but we were unable to identify this in the cyclostome descendents, indicating that it may have been lost secondarily. It may be that a Dp116/G-utrophin isoform generated from the single dystrophin/utrophin gene has assumed the role of DRP2.

The pared-down DGC component repertoire of the ascidians is rather surprising, given their widespread conservation across the invertebrate phyla. We detected only dystrophin, dystroglycan and dystrotelin in the two almost complete ascidian genome sequences. Although we cannot rule out the existence of rather divergent sarcoglycan sequences, we are confident that the ascidians, members of phylum chordata, lack both dystrobrevin and the syntrophins (sequences readily detectable in *D. melanogaster*, *C. elegans *and *S. mansoni*). In line with this observation, the binding sites for these proteins on the ascidian dystrophin molecule are highly degenerate, even compared with those from distant phyla. Furthermore, the dystrophin is N-terminally truncated, being slightly smaller than vertebrate Dp116. This is consistent with hypotheses and observations regarding the "streamlining" of ascidian biology [45,46], and also demonstrates that loss of some core components of the DGC can be compatible with metazoan life.

The discovery of the novel molecule γ-dystrobrevin is remarkable in several ways. First, its sequence reproducibly diverges *before *the divergence of fish and tetrapods in both the α- and β-dystrobrevin lineages (see Figure 5). The clear inference from this is that the last common ancestor of fish and tetrapods must have had three dystrobrevin paralogues, with our ancestors losing the γ-dystrobrevin gene at a later date. Second, in fish, the β-dystrobrevin gene has lost several exons in the central syntrophin-binding region, which the γ-dystrobrevin gene has retained (this may account for its common mis-annotation as β-dystrobrevin in the databases). Third, there are, apart from these missing exons, no obvious differences between the β- and γ-dystrobrevins; perhaps the salient differences are in their patterns of expression. An intriguing possibility is that rather than regulate syntrophin stoichiometry by alternative splicing of β-dystrobrevin exon 13 (as happens in mammals[9], but not fish), fish could achieve essentially the same end by alternative expression of β- and γ-dystrobrevin. Our preliminary analysis of the expression of this fish-specific dystrobrevin in *D. rerio *shows that its mRNA localises predominantly to the vertical myosepta, structures which have been the prime focus for the study of the dystrophin complex in fish [58-60]. Full-length dystrophin, dystroglycan, and several sarcoglycans have been shown to localise to the myosepta [58-60], as has dystrophin mRNA[32], although the mechanism and significance of the latter is not understood. It has been assumed that the vertical myosepta are homologues of tetrapod myomuscular and/or myotendinous junctions. Our finding of a specific gene encoding a myoseptal dystrobrevin may aid targeted manipulation of the dystrophin complex in muscle cells.

Dystrotelin was an unanticipated discovery, given the high level of scrutiny of the vertebrate genome sequences. It is likely to have evaded description because the similarity of individual exon sequences to dystrophin is barely significant and it is poorly represented in EST databases. There are several features of dystrotelin which prompt immediate interest. First, the existence of a novel distant relative of dystrophin and dystrobrevin, sharing common core structures of these proteins yet differing from them as much as they differ from each other (see Figure 5), obviously presents a novel opportunity for understanding the core function of the superfamily. Second, while dystrophin and dystrobrevin have been highly conserved throughout metazoan evolution, the more rapidly diverging sequence of dystrotelin bears testament to a very different level of functional constraint. A possible explanation for this lower conservation is that while dystrophin and dystrobrevin form the core of an intricate multiprotein complex, dystrotelin may act alone or in partnership with a smaller number of proteins. Ongoing interaction studies may help resolve this issue. It is particularly striking that despite broad conservation throughout phylum chordata (and possibly beyond), the chimpanzee appears not to have an intact dystrotelin gene. Third, it seems probable (though hard to prove) that dystrotelin is the vertebrate orthologue of DAH, a protein with an intriguing null phenotype in *Drosophila*. DAH is involved in the synchronised cellularisation of thousands of nuclei in the syncytial early fly embryo (a specialised form of cytokinesis); in its absence, the invaginating cleavage furrows which ultimately enfold the nuclei are severely disrupted, and development stalls[52]. DAH has been shown to be tightly membrane-associated and highly phosphorylated in a temporally regulated manner[61], and seems to be associated with vesicles which convene at the extending cleavage furrow[62]. Its localisation depends on NUF (nuclear fallout)[62], the fly orthologue of vertebrate pericentrosomal and cleavage furrow proteins Rab11-fip3 and Rab11-fip4. A vertebrate counterpart of the *Drosophila *cellularisation process is not obvious, although dystrotelin may be involved in other forms of cytokinesis. There is also the possibility that DAH has another function which is masked by the severity and early onset of the cellularisation defect. We surveyed the literature for candidate human diseases which might be associated defects in the dystrotelin gene; its position in chromosomal band 2q33.3 means that it is heterozygously deleted in several patients with complex but subtle phenotypes [63], and disorders such as early-onset osteoarthritis[64] and spastic ataxia[65] have recently been linked to markers nearby.

## Conclusion

By way of a summary, we can now sketch a broader picture of the evolution of the dystrophin/dystrobrevin/dystrotelin superfamily (see upper case letters in Figure 5).

A. The three branches of the superfamily (dystrophin, dystrobrevin and dystrotelin) originate from a single ancestral gene which had all the signature structural elements still borne by most representatives. We believe that it probably resembled β-dystrobrevins in gross structure. We are not able to resolve this trichotomy by phylogenetic means.

B. The dystrophin branch acquired a large N-terminal extension comprising CH domains, spectrin repeats and a WW domain. Although we cannot prove that this is an acquired trait through gene fusion, this explanation is more parsimonious than domain loss in the dystrobrevin and dystrotelin branches. SMART architecture analysis[66] reveals no extant proteins with this domain architecture from which we might speculate on the nature of the source of this material.

C. The affinity of the DAH family is uncertain, hence its emergence from a trichotomy on the tree. However we have advanced a number of circumstantial arguments that it is the orthologue of the dystrotelins, and that this affinity has been masked by the rapid rate of sequence change in this clade.

D. The invertebrate repertoire of single dystrophin, dystrobrevin and dystrotelin genes seems almost universal, except for ascidians.

E. The streamlined genome of the ascidians (sea squirts) lost several core components of the DGC, including dystrobrevin and both syntrophins; it retains dystroglycan, dystrophin and dystrotelin.

F. The Dp116 region of the single invertebrate dystrophin gene underwent a duplication in an ancestor of all craniates to give rise to DRP2 and a common ancestor of dystrophin and utrophin.

G. At a similar time, the invertebrate dystrobrevin gene underwent a tandem duplication of exon 14 to give the vertebrate-specific exon 13, and the entire gene was duplicated to give rise to γ-dystrobrevin and a common ancestor of α- and β-dystrobrevin.

H. The dystrophin/utrophin gene in an ancestor of all gnathostomes acquired exon 71 then underwent a whole-gene duplication to give rise to separate dystrophin and utrophin genes, including paralogous short isoforms Dp116 and G-utrophin. The dystrophin gene acquired exon 78 which allows it to conditionally remove the C-terminus of the protein.

I. DRP2 and the C-terminus of dystrophin/utrophin were lost in an ancestor of extant cyclostomes.

J. The α/β-dystrobrevin gene underwent a whole-gene duplication in an ancestor of all gnathostomes to give rise to separate α- and β-dystrobrevin genes. The α-dystrobrevin gene gained exons 9 and 12 and those encoding the novel C-terminus, while the β-dystrobrevin gene of fish lost exons 10 and 13.

K. An ancestor of all tetrapods gained novel intragenic promoters for the Dp260 and Dp140 isoforms in the dystrophin gene, and lost the γ-dystrobrevin gene.

L. Point mutations resulted in the functional loss of exons 11 and 12 in primate dystrotelin genes and disrupted the ORF in chimpanzee.

Basal vertebrates therefore have a repertoire of seven superfamily members (three dystrophins, three dystrobevins, and one dystrotelin), with one of these being lost in tetrapods. Most invertebrates studied have three members, one from each branch. The basic shared function which is implied by the common architecture of these distantly related proteins remains unclear, but it is our hope that this broadening of the scope of dystrophin biology will afford ever more avenues of investigation to bear on its elucidation.

## Methods

### RNA and DNA preparation

Dissected tissues or whole animal samples from mouse (*Mus musculus*), rat (*Rattus norvegicus*), frog (*Xenopus laevis*), zebrafish (*Danio rerio*), shark (*Scyliorhinus canicula*), hagfish (*Myxine glutinosa*), lamprey (*Petromyzon marinus*, *Lampetra planeri*), and schistosomes (*Schistosoma mansoni*) were homogenised in Trizol reagent (Invitrogen) and RNA was extracted according to manufacturers' instructions. Genomic DNA was prepared from CRL-1609 chimpanzee (*Pan troglodytes*) fibroblasts, MLA-144 gibbon (*Hylobates lar*) T cell line, and shark (*S. canicula*) liver.

### RT-PCR, RACE and sequence analysis

Nested RT-PCR[15], semi-quantitative RT-PCR[4], 5'cRACE[23], and 3'RACE combined with vectorette ligation[30] were performed as previously described. Products were either gel-purified using QiaQuick columns (Qiagen) and sequenced directly or TA-cloned into pCR4-TOPO vector (Invitrogen), after which plasmid DNA was prepared using a Qiaprep plasmid miniprep kit (Qiagen). Sequencing was performed using BigDye Terminator v3.1 Cycle Sequencing Kit (Applied Biosystems, Inc.) and BetterBuffer (Microzone Ltd) according to manufacturers' instructions, followed by electrophoretic analysis on an Applied Biosystems 3730 DNA Analyser. All oligonucleotides were synthesised by MWG-Biotech ([see Additional File 4]; degenerate oligonucleotides previously described [15]). Quantitation of agarose gels stained with ethidium bromide was done using a Typhoon 9200 Imager (GE Healthcare) with ImageQuant software.

### Heterologous expression and fluorescent microscopy

For analysis of subcellular localisation of dystrotelin, the coding region (1951 bp, including 35 bp of 5'UTR and codons 1–639) of mouse dystrotelin was ligated into the *Hin*dIII and *Bam*HI sites of pEGFP-N2 (Clontech), such that it was in-frame with the EGFP coding sequence. The construct was transfected into cultured *Cercopithicus aethiops *COS-7 cells using Fugene 6 transfection reagent (Roche) according to the manufacturers' instructions. Cells were counterstained for nuclei using Vectashield mounting medium with DAPI (Vector Laboratories, Inc.), for mitochondria using Mito Tracker Red CMXRos (Molecular Probes), and for endoplasmic reticulum using ER-Tracker Red (Invitrogen). Fluorescent images were captured using a Zeiss LSM510 confocal fluorescent microscope.

### Whole mount mouse *in situ *hybridization

Mouse day E10.5 embryos were fixed in 4% paraformaldehyde and subjected to *in situ *hybridisation according to Mootoosamy & Dietrich[67]. The probe was generated from a 673-bp cDNA encoding amino acids 24–248 of mouse dystrotelin.

### Whole mount zebrafish *in situ *hybridization

26 hpf Zebrafish Embryos were fixed in 4% paraformaldehyde, stored in methanol at 20°C and processed for *in situ *mRNA hybridisation as described[68,69]. Antisense probes or sense controls were synthesised from linearised pCR4 plasmids with T7 or T3 polymerase (Roche) using digoxigenin-labelling mix (Roche). The probes were generated from a 419-bp cDNA of the 3'UTR of dystrophin and a 764-bp cDNA of the 3'UTR of γ-dystrobrevin.

### Yeast two-hybrid analysis

Yeast two-hybrid analysis was carried out as previously described[70], co-transforming pBHA-based bait constructs and pGAD10-based prey constructs into L40 strain *S. cerevisiae*, and assessing interaction by the activation of *LacZ *and HIS3 reporter genes (detected by ability to hydrolyse X-gal and ability to grow on histidine-depleted minimal medium, respectively). Constructs contained sequences encoding amino acids 258–654 of mouse dystrotelin, 3484–3600 of mouse dystrophin, or 461–554 of mouse α-dystrobrevin, cloned into the *Eco*RI and *Sal*I sites of pBHA and the *Xho*I and *Eco*RI sites of pGAD10.

### Bioinformatics

Genomic sequences were acquired from the Ensembl Genome Server[71] and other sequences from the databases at NCBI[72]. Sequences were curated and manipulated in BioEdit[73] and Vector NTI Suite (InforMax, Inc.). Most phylogenetic analysis was performed using programs from the Phylip package(.)[74] within the BioEdit platform. Ka/Ks calculations were performed using the FUGE bioinformatics platform[75,76]. Coiled-coil regions were predicted using COILS[77,78], and protein secondary structure was predicted using PSIPRED[49,79]. Dotplots were performed using DOTTER[80]. Some analysis of domain architecture was done using SMART[66].

### New GenBank accession numbers

DQ431250-1, DQ440977-9, DQ443726-8, DQ516343-7, DQ640072-3, DQ641922, DQ788695-6, DQ831004-6, U43517 (updated), BK005803.

## Abbreviations

Central nervous system (CNS), peripheral nervous system (PNS), polymerase chain reaction (PCR), reverse transcript PCR (RT-PCR), expressed sequence tag (EST), Duchenne muscular dystrophy (DMD), open reading frame (ORF), untranslated region (UTR), dystrophin-related protein 2 (DRP2), rapid amplification of cDNA ends (RACE), discontinuous actin hexagon (DAH), dystrophin-glycoprotein complex (DGC), ribonucleic acid (RNA), deoxyribonucleic acid (DNA), yeast two-hybrid (Y2H), enhanced green fluorescnet protein (EGFP), endoplasmic reticulum (ER), hours post fertilisation (hpf).

## Authors' contributions

HJ acquired dystrobrevin and dystrophin isoform sequences, and performed yeast two-hybrid analysis and heterologous expression experiments. ST acquired DRP2, cyclostome and dystrotelin sequences and generated the dystrotelin expression profile. JH acquired dystrotelin sequences and generated constructs for other experiments. SB, YH and SMH performed *in situ *hybridisation on zebrafish. SP and PTS performed whole-mount *in situ *hybridisation on mouse embryos. JMM established the temporal pattern of alternative splicing in fish. MJG did some of the earlier analyses of cyclostome sequence. RGR conceived the project, performed most of the bioinformatic analysis, and drafted the manuscript. All authors read and approved the final manuscript.

## Supplementary Material

Additional File 1 **Sequences of alternative first exons of the dystrophin and utrophin genes**. Alignments of: A) N-terminal translation of Dp260 transcript (Dp260 first exon and exon 30), B) first exon and part of exon 45 of Dp140 transcript, C) N-terminal translation of Dp116 transcript (Dp116 first exon and exon 56) and G-utrophin transcript (G-utrophin first exon and exon 56), D) N-terminal translation of Dp71 transcript (Dp71 first exon and exons 63 and 64). Vertical lines denote exon boundaries. Accession numbers: Dp260, *H. sapiens *[GenBank:U27203], *M. musculus *[GenBank:CX568898], *X. laevis *[GenBank:DQ831004]; Dp140, *H. sapiens *[GenBank:L35854], *M. musculus *[GenBank:CJ185327], *X. laevis *[GenBank:DQ831006]; Dp116, *H. sapiens *[GenBank:S62617], *M. musculus *[GenBank:AK149002], *R. norvegicus *[GenBank:S76214], *D. rerio *[GenBank:DQ788695]; G-utrophin, *H. sapiens *[GenBank:BP282211], *M. musculus *[GenBank:X83506], *R. norvegicus *[GenBank:CB546060], *D. rerio *[GenBank:DQ788696], *D. rerio *pseudogene [GenBank:DQ831005]; Dp71, *H. sapiens *[GenBank:M92650], *Canis familiaris *[GenBank:AY566609], *M. musculus *[GenBank:S62620], *R. norvegicus *[GenBank:X69767], *G. gallus *[GenBank:CN223681], *X. laevis *[GenBank:BC082429]*, D. rerio *[GenBank:AF339032], *Squalus acanthias *[GenBank:CV798132]. Sequences without accession numbers are predicted from genomic sequence alone. Click here for file

Additional File 2 **Pyrimidine +3 and alternative splicing of vertebrate dystrophin and dystrobrevin transcripts**. A) Alignment of genomic context of dystrophin exon 78, which shows conservation of alternative splicing across vertebrates. Arrow shows the unusual pyrimidine at position +3. B) Alignment of genomic context of exon 13 of the vertebrate dystrobrevin exon 13, which shows alternative splicing across vertebrates in the α- and β-dystrobrevins, but not the γ-dystrobrevins. Arrow shows that a pyrimidine occurs at position +3 in the genes that undergo alternative splicing; this is replaced by a purine in those genes (the γ-dystrobrevins) which constitutively include exon 13. All sequences derived from Ensembl genome databases except *S. canicula *exon 78 (this work) [GenBank:DQ641922]. Upper case and yellow boxes – exons; lower case – introns. Click here for file

Additional File 3 **High Ka/Ks values in the dystrotelin family**. Ka/Ks values for the six main vertebrate dystrophin-related proteins (γ-dystrobrevin is only found in fish). The three columns show the degree of pressure to conserve amino acid sequence within rodents, mammals and vertebrates, respectively. High Ka/Ks values suggest weak negative selection or partial positive selective pressures acting on dystrotelin, particularly in the C-terminal region (shaded). All other family members show Ka/Ks < 0.2, indicative of strong purifying selection acting at the amino acid level. Click here for file

Additional File 4 **Oligonucleotide primers used in this study**. Not all primers are given, but these represent the majority of those used for RT-PCR and genomic PCR. Sequences of primers used for internal sequencing and construct generation are available on request. Click here for file
