# Supplementary material for: The dystrotelin, dystrophin and dystrobrevin superfamily: new paralogues and old isoforms
Source: BMC Genomics. 2007 Jan 17;8:19. doi: 10.1186/1471-2164-8-19 (PMC1790709; doi:10.1186/1471-2164-8-19)
Supplement: Additional File 1 — Sequences of alternative first exons of the dystrophin and utrophin genes. Alignments of: A) N-terminal translation of Dp260 transcript (Dp260 first exon and exon 30), B) first exon and part of exon 45 of Dp140 transcript, C) N-terminal translation of Dp116 transcript (Dp116 first exon and exon 56) and G-utrophin transcript (G-utrophin first exon and exon 56), D) N-terminal translation of Dp71 transcript (Dp71 first exon and exons 63 and 64). Vertical lines denote exon boundaries. Accession numbers: Dp260, H. sapiens [GenBank:U27203], M. musculus [GenBank:CX568898], X. laevis [GenBank:DQ831004]; Dp140, H. sapiens [GenBank:L35854], M. musculus [GenBank:CJ185327], X. laevis [GenBank:DQ831006]; Dp116, H. sapiens [GenBank:S62617], M. musculus [GenBank:AK149002], R. norvegicus [GenBank:S76214], D. rerio [GenBank:DQ788695]; G-utrophin, H. sapiens [GenBank:BP282211], M. musculus [GenBank:X83506], R. norvegicus [GenBank:CB546060], D. rerio [GenBank:DQ788696], D. rerio pseudogene [GenBank:DQ831005]; Dp71, H. sapiens [GenBank:M92650], Canis familiaris [GenBank:AY566609], M. musculus [GenBank:S62620], R. norvegicus [GenBank:X69767], G. gallus [GenBank:CN223681], X. laevis [GenBank:BC082429], D. rerio [GenBank:AF339032], Squalus acanthias [GenBank:CV798132]. Sequences without accession numbers are predicted from genomic sequence alone. [file 1471-2164-8-19-S1.doc]

A. Dystrophin Dp260

Dp260 E1

E30

**Human MSARKLRNLSYKKAVRRQKLLEQSIQSAQETEKSLHLIQESL**

**Mouse MSARKLRNLSYKKAVRKQKLLEQSIQSAQEIEKSLHLIQESL**

**Chicken** **MSGKKMRNLSYKKAVRRQKSLEQSIQSAQETDKTLRLIQESL**

**Frog MNSKSHRNSSYKKAVRRQKVLEQSIQSAQETDKAIRMIQEAL**

B. Dystrophin Dp140

Dp140 E1

E45

**Human actagcaatggcaaagctttgtgcggaggcattgctggctgctctgaactaaaagcatccgtggggaccgaaagaggtttttgcacac---cttattaaggaactccaggatggcattggg**

**Mouse actagcaatggcaaagctttgtgcggaggcattgctgactgttctgagctaaaatcgtcagtgtggaccagaagggggttttgcacac---cttattaaggaactccaggatggcattggg**

**Chicken** **actcgcaatggcaaagctttgtgcggatgcgctgctggatgcgctgagctaaaagcgtc-ggggggagcggagcgcggttcgggctgc---cttttaaaggagcttcaggatggcattggg**

**Frog actagcaatggcaaagctctgtacggatgcactgctggattctctgaactaatatcatttgtgggacgttaaaaagattcccttgaacgtcatctttaaggagattgaagatggcatagga**

C. Dystrophin Dp116, G-Utrophin

Dp116 E1

E56

**Human ----MQQDQCCSARFKLKMLHR-KTYHVK-----------------------DLQGEIEAHTDV**

**Rat ----MQQDQCCSPRFKLKMLHR-KTYHVK-----------------------DLQGEIEAHTDI**

**Mouse ----MQQDQCCSPRFKLKMLHR-KTYHVK-----------------------DLQGEIETHTDI**

Dp116

**Opossum ----MQQDQCCSPRFKLKMLHR-KTYHVK-----------------------DLQGEIEAHTDI**

**Chicken ----MQQEQCFG-QLKLKILHR-KSLRGK-----------------------DLQAEIDAHTDI**

**Frog ------------------MLHR-KKYNVK-----------------------NLQEEIDGHTEV**

**Zebrafish ------------------MLHR-KTYHVK-----------------------DLQAEIDAHRET**

**Human MQIL-RCLQKCG---KLKMMAVVRTSLQKVVVLLHRLQRMAVSSPRYQKLCKDIQAEIDAHNDI**

**Mouse MQIL-RTLQKCG---KLKMMAVVRTSLQKVVVLLHRLQRMAVSSPRYQKLCKDIQAEIDAHNDI**

**Rat MQIL-RTLQKCG---KLKMMAVVRTSLQKVVVLLHRLQRMAVSSPRYQKLCKDIQAEIDAHNDI**

**Chicken MQIL-RCLQKCG---KLKMMAVVRTSLQKVVVLLHRLQRMAVSSPRYQKLCKDIQAEIDAHNDI**

# G-utrophin

**Frog MQIL-RCLQKCG---KLKMMAVVRTSLQKVVVLLHHLQRMAVSSPRYQKLCKDIQAEIDAHNDI**

**Zebrafish MRAL-RRWQSCAG--RVRMMAVVRTSLQKVVVFLHRLQRMAISSPRYQKLCKDIQAEIEAHNDV**

**Fugu MRTKSRLLQTCS---SLKMMAMVRTSLQKVVVFLHRLQRMAISSPRYQKLCKDIQAEIDAHNDI**

**Zebrafish ψ** **MRVLDKPSS-------LKIMAMVRSSLQKVMLFLQHIQRMAVTSPRYQKLCKDIQTDVDTQTDL**

G-Utrophin E1

E56

D. Dystrophin Dp71

Dp71 E1

E63

E64

**Human MREQLKGHETQTTCWDHPKMTELYQSLADLNNVRFSAYRTAM**

**Dog MREQLKGHETQTTCWDHPKMTELYQSLADLNNVRFSAYRTAM**

**Mouse MREHLKGHETQTTCWDHPKMTELYQSLADLNNVRFSAYRTAM**

**Rat MREHLKGHETQTTCWDHPKMTELYQSLADLNNVRFSAYRTAM**

**Chicken MREQRRGHETQTTCWDHPKMTELYQSLADLNNVRFSAYRTAM**

**Frog MRELLKGHETQTTCWDHPKMTELYQSLADLNNVRFSAYRTAM**

**Zebrafish** **MRENLRNHQTQTTCWDHPKMAELYQSLADLNNVRFSAYRTAM**

**Shark MREQLKGHQTQTTCWDHPKMTELYQSLADLNNVRFSAYRTAM**
