# Supplementary material for: The dystrotelin, dystrophin and dystrobrevin superfamily: new paralogues and old isoforms
Source: BMC Genomics. 2007 Jan 17;8:19. doi: 10.1186/1471-2164-8-19 (PMC1790709; doi:10.1186/1471-2164-8-19)
Supplement: Additional File 2 — Pyrimidine +3 and alternative splicing of vertebrate dystrophin and dystrobrevin transcripts. A) Alignment of genomic context of dystrophin exon 78, which shows conservation of alternative splicing across vertebrates. Arrow shows the unusual pyrimidine at position +3. B) Alignment of genomic context of exon 13 of the vertebrate dystrobrevin exon 13, which shows alternative splicing across vertebrates in the α- and β-dystrobrevins, but not the γ-dystrobrevins. Arrow shows that a pyrimidine occurs at position +3 in the genes that undergo alternative splicing; this is replaced by a purine in those genes (the γ-dystrobrevins) which constitutively include exon 13. All sequences derived from Ensembl genome databases except S. canicula exon 78 (this work) [GenBank:DQ641922]. Upper case and yellow boxes – exons; lower case – introns. [file 1471-2164-8-19-S2.ppt]

## Slide 1
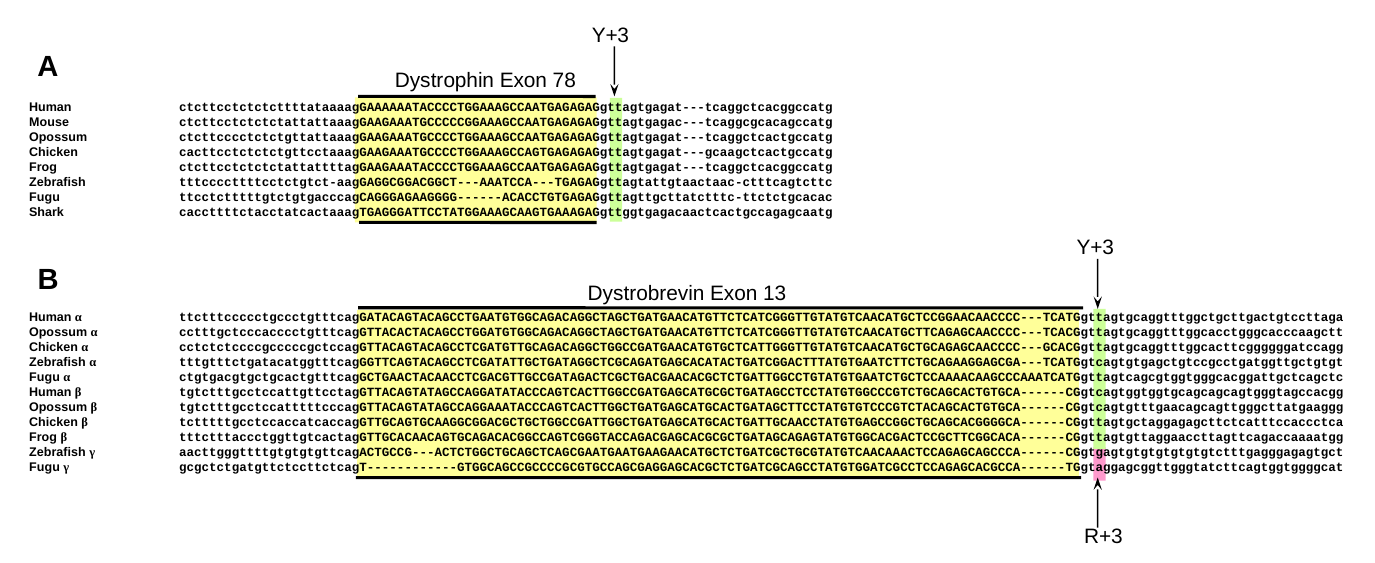

Human	ctcttcctctctcttttataaaagGAAAAAATACCCCTGGAAAGCCAATGAGAGAGgttagtgagat---tcaggctcacggccatg
Mouse	ctcttcctctctctattattaaagGAAGAAATGCCCCCGGAAAGCCAATGAGAGAGgttagtgagac---tcaggcgcacagccatg
Opossum	ctcttcccctctctgttattaaagGAAGAAATGCCCCTGGAAAGCCAATGAGAGAGgttagtgagat---tcaggctcactgccatg
Chicken	cacttcctctctctgttcctaaagGAAGAAATGCCCCTGGAAAGCCAGTGAGAGAGgttagtgagat---gcaagctcactgccatg
Frog	ctcttcctctctctattattttagGAAGAAATACCCCTGGAAAGCCAATGAGAGAGgttagtgagat---tcaggctcacggccatg
Zebrafish	tttccccttttcctctgtct-aagGAGGCGGACGGCT---AAATCCA---TGAGAGgttagtattgtaactaac-ctttcagtcttc
Fugu	ttcctctttttgtctgtgacccagCAGGGAGAAGGGG------ACACCTGTGAGAGgttagttgcttatctttc-ttctctgcacac
Shark	caccttttctacctatcactaaagTGAGGGATTCCTATGGAAAGCAAGTGAAAGAGgttggtgagacaactcactgccagagcaatg
Human α	ttctttccccctgccctgtttcagGATACAGTACAGCCTGAATGTGGCAGACAGGCTAGCTGATGAACATGTTCTCATCGGGTTGTATGTCAACATGCTCCGGAACAACCCC---TCATGgttagtgcaggtttggctgcttgactgtccttaga
Opossum α	cctttgctcccacccctgtttcagGTTACACTACAGCCTGGATGTGGCAGACAGGCTAGCTGATGAACATGTTCTCATCGGGTTGTATGTCAACATGCTTCAGAGCAACCCC---TCACGgttagtgcaggtttggcacctgggcacccaagctt
Chicken α	cctctctccccgcccccgctccagGTTACAGTACAGCCTCGATGTTGCAGACAGGCTGGCCGATGAACATGTGCTCATTGGGTTGTATGTCAACATGCTGCAGAGCAACCCC---GCACGgttagtgcaggtttggcacttcggggggatccagg
Zebrafish α	tttgtttctgatacatggtttcagGGTTCAGTACAGCCTCGATATTGCTGATAGGCTCGCAGATGAGCACATACTGATCGGACTTTATGTGAATCTTCTGCAGAAGGAGCGA---TCATGgtcagtgtgagctgtccgcctgatggttgctgtgt
Fugu α	ctgtgacgtgctgcactgtttcagGCTGAACTACAACCTCGACGTTGCCGATAGACTCGCTGACGAACACGCTCTGATTGGCCTGTATGTGAATCTGCTCCAAAACAAGCCCAAATCATGgttagtcagcgtggtgggcacggattgctcagctc
Human β	tgtctttgcctccattgttcctagGTTACAGTATAGCCAGGATATACCCAGTCACTTGGCCGATGAGCATGCGCTGATAGCCTCCTATGTGGCCCGTCTGCAGCACTGTGCA------CGgtcagtggtggtgcagcagcagtgggtagccacgg
Opossum β	tgtctttgcctccatttttcccagGTTACAGTATAGCCAGGAAATACCCAGTCACTTGGCTGATGAGCATGCACTGATAGCTTCCTATGTGTCCCGTCTACAGCACTGTGCA------CGgtcagtgtttgaacagcagttgggcttatgaaggg
Chicken β	tctttttgcctccaccatcaccagGTTGCAGTGCAAGGCGGACGCTGCTGGCCGATTGGCTGATGAGCATGCACTGATTGCAACCTATGTGAGCCGGCTGCAGCACGGGGCA------CGgttagtgctaggagagcttctcatttccaccctca
Frog β	tttctttaccctggttgtcactagGTTGCACAACAGTGCAGACACGGCCAGTCGGGTACCAGACGAGCACGCGCTGATAGCAGAGTATGTGGCACGACTCCGCTTCGGCACA------CGgttagtgttaggaaccttagttcagaccaaaatgg
Zebrafish γ	aacttgggttttgtgtgtgttcagACTGCCG---ACTCTGGCTGCAGCTCAGCGAATGAATGAAGAACATGCTCTGATCGCTGCGTATGTCAACAAACTCCAGAGCAGCCCA------CGgtgagtgtgtgtgtgtgtctttgagggagagtgct
Fugu γ	gcgctctgatgttctccttctcagT------------GTGGCAGCCGCCCCGCGTGCCAGCGAGGAGCACGCTCTGATCGCAGCCTATGTGGATCGCCTCCAGAGCACGCCA------TGgtaggagcggttgggtatcttcagtggtggggcat
Y+3
A
Dystrophin Exon 78
Y+3
B
Dystrobrevin Exon 13
R+3
