# Supplementary material for: The dystrotelin, dystrophin and dystrobrevin superfamily: new paralogues and old isoforms
Source: BMC Genomics. 2007 Jan 17;8:19. doi: 10.1186/1471-2164-8-19 (PMC1790709; doi:10.1186/1471-2164-8-19)
Supplement: Additional File 3 — High Ka/Ks values in the dystrotelin family. Ka/Ks values for the six main vertebrate dystrophin-related proteins (γ-dystrobrevin is only found in fish). The three columns show the degree of pressure to conserve amino acid sequence within rodents, mammals and vertebrates, respectively. High Ka/Ks values suggest weak negative selection or partial positive selective pressures acting on dystrotelin, particularly in the C-terminal region (shaded). All other family members show Ka/Ks < 0.2, indicative of strong purifying selection acting at the amino acid level. [file 1471-2164-8-19-S3.doc]

|  | Ka/Ks Mouse vs | | |
| --- | --- | --- | --- |
|  | Rat | Human | Fish^a^ |
| Dystrophin | NA^b^ | 0.01 | 0.06 |
| Utrophin | 0.04 | 0.03 | 0.13^c^ |
| DRP2 | 0.05 | 0.05 | 0.18 |
| α-Dystrobrevin | 0.07 | 0.06 | 0.18 |
| β-Dystrobrevin | 0.07 | 0.06 | 0.09 |
| γ-Dystrobrevin | NA^d^ | NA^d^ | NA^d^ |
| Dystrotelin (1-642) | 0.62 | 0.66 | 0.67 |
| Dystrotelin (1-277) | 0.37 | 0.37 | 0.44 |
| Dystrotelin (278-642) | 0.93 | 0.97 | NA^e^ |

^a^Zebrafish (*Danio rerio*) except where stated.

^b^Ka=0.

^c^Lesser spotted catshark (*Scyliorhinus canicula*).

^d^γ-Dystrobrevin not present in mammals

^e^Fish sequence not alignable.
