# Supplementary material for: The dystrotelin, dystrophin and dystrobrevin superfamily: new paralogues and old isoforms
Source: BMC Genomics. 2007 Jan 17;8:19. doi: 10.1186/1471-2164-8-19 (PMC1790709; doi:10.1186/1471-2164-8-19)
Supplement: Additional File 4 — Oligonucleotide primers used in this study. Not all primers are given, but these represent the majority of those used for RT-PCR and genomic PCR. Sequences of primers used for internal sequencing and construct generation are available on request. [file 1471-2164-8-19-S4.doc]

**Dystrophin/Utrophin promoters:**

ZebDp116FO: cgtttgatgccagtcaatcacc

ZebDp116FI: atcaccggatatgttgcacagg

ZebDp116RO: ccctcttcagctgcagccagttg

*D. rerio* dystrophin Dp116

ZebDp116RI: gttgagcagctcttgtaaggac

ZebGUtrFO: gtgaggatgcgtgctctcagac

*D. rerio* G-utrophin

ZebGUtrFI: cagacgctggcagtcgtgtgc

ZebGUtrRO: cagtaatgtgggcacgtctcctc

ZebGUtrRI: ctttcagaccgatccagcggctc

XenDp260FO: atccagcagcctaaatgaattc

*X. tropicalis/X. laevis* dystrophin Dp260

XenDp260FI: aaagtcatagaaattcatcctac

XenDp260RO: gctgcgtctgaacaacttcctg

XenDp260RI: ccaaaactggcaattcgaatgtc

XenDp140FO: ggatgcactgctggattctctg

XenDp140FI: ctaatatcatccgtgagacc

*X. tropicalis/X. laevis* dystrophin Dp140

XenDp140RO: ttatccagcgctggttggcctc

XenDp140RI: ctaatgccgttcctccagcttc

**Dystrotelins:**

HumChiOthE4F: ctaggtataggataagaggccac

*H. sapiens/P. troglodytes*/*H. lar* dystrotelin genomic

HumChiOthE4R: gctgaagaggccttgtggcaggg

HumChiOthE5F: cctttcatagccacaaagagactg

HumChiOthE5R: gacttgacactggagacatcag

HumChiOthE6F: atacttgtgactacacagccgtg

HumChiOthE6R: gttatctgagatgctatgtggag

HumOthEZRO: ggccctgcacactcgctgag

HumOthEZRI: ctgagctccactgtacaggtcc

*H. sapiens* dystrotelin cDNA

HumOthEZRO2: gtcatcacaccaagaggcctttgag

HumOthEZRI2: gagcctggactccatttcacttc

HumOthE7FO: cctgccaccggttatcagctgc

HumOthE7FI: cactcaccctgctcggtgcactc

HumOthE1FO: cagggacccacttgccatctgtc

HumOthE1FI: gatccagataaacaagatgctcttaatagtattgagaattcc

HumOthE8RO: ctgaatgcagtgctcaatgac

HumOthE8RI: ctgaatgcagtgctcaatgacaggatgag

HsOTH-E9FO: gcagcagctgctggaccaggtg

HsOTH-E9FI: ggctgttcctgtgcaacaatg

MusOthEZRO: cagagaaggccctgcacacctg

MusOthEZRI: gggctctaccatacagctcc

# MmOTH-E2FO: tctgtctatagaacagccttc

*M. musculus* dystrotelin cDNA

# MmOTH-E2FI: cgatctgtgcaaactctttgcc

# MmOTH-E9RO: ctggtccagcagccactgtc

# MmOTH-E9RI: ggttgtttctgagactcctg

# MmOTH-E8FO: accattgtctaaagtgcctgg

# MmOTH-E8FI: ctggtctccacaaaaattccc

ZebOthE1FO: cagtttatgcgagcagcagac

ZebOthE1FI: gcgagcagcagactgttatgg

ZebOthE8RO: ctgagtgcagtactccagcac

ZebOthE8RI: ctccagcacagaatgggaggg

*D. rerio* dystrotelin cDNA

ZebOTH-E14RO: aattagtgcaaatgtgaggcacag

ZebOTH-E14RI: ccatcatgccaaactggagctc

ZebOthE7FO: ggctgtccacactctacaggatttc

ZebOthE7FI: gttcactgccatgcctgtaaagcc

**Fish DRP2:**

ZebDRP2-5RT: P-cccgacgtctcctccgatgggcagc

ZebDRP2-5FO: caacatcagggtacggcttgaggc

*D. rerio* dystrotelin cDNA

ZebDRP2-5FI: cagcgggaagctccagctgtccc

ZebDRP2-5RO: cctgcactgggtgggcagctcctg

ZebDRP2-5RI: ccctcaggatccaccgccgactg

**Fish Dystrobrevins:**

ZebAllDybFO: ggntttcgstaycgmtgycagcagtg

ZebAllDybRO: carrcgctgratctcytgcarratctc

ZebDybA-ISFO: ctcctggtagctgcagactcc

ZebDybA-ISFI: ccaacacaatgtcctcactggtc

*D. rerio* α-dystrobrevin cDNA

ZebDybA-ISRO: acgaacgaacgcagtaacatc

ZebDybA-ISRI: ctgtatttatggcactggcgg

ZebADybFI: gtaaatctctgagctgtgcggcgag

ZebADybRI: ctgtttattagagtccaagctg

ZebDybB-ISFO: cttttagtggctgcagactcg

ZebDybB-ISFI: cactagtgaaagaacttcactc

ZebDybB-ISRO: aggcttcactcgagtcgctcg

*D. rerio* β-dystrobrevin cDNA

ZebDybB-ISRI: gcctctccaccaatccagcag

ZebBDybFI: aatctctgggctgcatgccgatc

ZebBDybRI: ctgctgcttattagcatcaaagttg

ZebDybG-ISFO: gaaagactgttgcagaatggc

ZebDybG-ISFI: tggcaaagacagaggttaggc

ZebDybG-ISRO: acagcaggtcagatgtgtgcg

*D. rerio* γ-dystrobrevin cDNA

ZebDybG-ISRI: ttcacaggaaacagtgactac

ZebGDybFI: aagtctttgggttgtgtgccgag

ZebGDybRI: gctgtttgttggtgtcgtagctg
